# Diabetes Promotes Retinal Vascular Endothelial Cell Injury by Inducing CCN1 Expression

**DOI:** 10.3389/fcvm.2021.689318

**Published:** 2021-08-11

**Authors:** Haicheng Li, Ting Li, Heting Wang, Xuemin He, Ying Li, Siying Wen, Rongdong Peng, Yuanpeng Nie, Yan Lu, He Yang, Yinong Ye, Guojun Shi, Yanming Chen

**Affiliations:** ^1^Department of Endocrinology and Metabolism, The Third Affiliated Hospital of Sun Yat-Sen University, Guangzhou, China; ^2^Cancer Science Institute of Singapore, National University of Singapore, Singapore, Singapore; ^3^Department of Clinical Immunology, The Third Affiliated Hospital of Sun Yat-Sen University, Guangzhou, China; ^4^Foshan Fourth People's Hospital, Foshan, China

**Keywords:** diabetic retinopathy, CCN1, oxidative stress, NOX4 NADPH oxidase, tight junction

## Abstract

**Purpose:** Diabetic retinopathy (DR) is one of the most common diabetic microvascular complications. However, the pathogenesis of DR has not yet been fully elucidated. This study aimed to discover novel and key molecules involved in the pathogenesis of DR, which could potentially be targets for therapeutic DR intervention.

**Methods:** To identify potential genes involved in the pathogenesis of DR, we analyzed the public database of neovascular membranes (NVMs) from patients with proliferative diabetic retinopathy (PDR) and healthy controls (HCs) (GSE102485, https://www.ncbi.nlm.nih.gov/geo/query/acc.cgi?acc=GSE102485). Further, we compared these findings by performing RNA-sequencing analysis of peripheral blood mononuclear cells (PBMC) from patients with DR, control patients with non-complicated diabetes mellitus (DMC), and HCs. To determine the critical role of candidate genes in DR, knockdown or knockout was performed in human retinal vascular endothelial cells (HRVECs). The oxidative stress pathway, as well as tight junction integrity, was analyzed.

**Results:** Transcriptional profiles showed distinct patterns between the NVMs of patients with DR and those of the HCs. Those genes enriched in either extracellular matrix (ECM)-receptor interaction or focal adhesion pathways were considerably upregulated. Both pathways were important for maintaining the integrity of retinal vascular structure and function. Importantly, the gene encoding the matricellular protein CCN1, a key gene in cell physiology, was differentially expressed in both pathways. Knockdown of CCN1 by small interfering RNA (siRNA) or knockout of CCN1 by the CRISPR-Cas9 technique in HRVECs significantly increased the levels of VE-cadherin, reduced the level of NADPH oxidase 4 (NOX4), and inhibited the generation of reactive oxygen species (ROS).

**Conclusion:** The present study identifies CCN1 as an important regulator in the pathogenesis of DR. Increased expression of CCN1 stimulates oxidative stress and disrupts tight junction integrity in endothelial cells by inducing NOX4. Thus, targeting the CCN1/NOX4 axis provides a therapeutic strategy for treating DR by alleviating endothelial cell injury.

## Introduction

Both diabetes (1) and pre-diabetes (2, 3) could lead to microvascular and macrovascular complication. Diabetic retinopathy (DR) is the most common complication of diabetes mellitus (DM); it has long been recognized as a microvascular disease (4) that affects nearly one-third of all patients with diabetes (5) and is the leading cause of vision loss globally (6). The condition results in a poor quality of life and increases mortality in patients. Etiological therapy is paramount for the management of DR and improvement of the prognosis. However, the pathogenesis of DR is not yet thoroughly understood. Thus, to date, no specific therapy is available to effectively treat DR.

The retina is the most metabolically active human tissue, and it is highly sensitive to hypoxia (7) DR has been shown to be closely related to hypoxia (8). Previous studies (9–11) have shown that CCN1, the first cloned member of the CCN family (12), is upregulated by hypoxia in several different cell types, including retinal vascular endothelial cells, along with the concurrent regulation of the transcriptional factors HIF-1a and c-Jun/AP-1(13). The CCN family consists of six distinct members, named according to the acronyms of the first three members: cysteine-rich (CYR61), connective tissue growth factor (CTGF), and nephroblastoma overexpressed (NOV) (14). They regulate cellular processes such as adhesion, migration, mitogenesis, differentiation, and survival (12). In addition to hypoxia, CCN1 expression in rat retinas is increased by more than 3-fold after 6 weeks of diabetes, and increased by 4-fold in the advanced glycation end product (AGE)-treated mouse retina (15). This indicates that the expression of CCN1 is positively correlated with the occurrence of DR.

Angiogenesis and fibrosis are the main characteristics of DR. The CCN1 family has been associated with both the fibrotic processes (16) and neovascularization of DR (17). According to one report, CCN1 induces fibroblast senescence and restricts cutaneous fibrosis; thus, tissue healing possibly occurs through the reactive oxygen species (ROS)-dependent activation of the p16INK4a/pRb pathway (18). The loss of retinal pericytes is another distinctive feature of DR, which is characterized by retinal capillary obliteration. Furthermore, CCN1 has been proven to induce pericyte detachment and anoikis (19). Previous studies suggest that CCN1 is involved in the pathogenesis of DR; however, the precise mechanism is still unknown.

The ROS are associated with one of the leading pathogenic mechanisms during DR progression. The NADPH oxidase (NOX) family produces ROS, which causes oxidative injuries and stimulates neovascularization in the retinal vasculature (20). In addition, NOX4 is the most abundant isoform in vascular endothelial cells (21). One study has shown that NOX4-generated ROS induces neuronal and blood-brain barrier injury after intracerebral hemorrhage (22). Our previous study (23) also demonstrated that NOX4 can stimulate inflammation in human retinal vascular endothelial cells (HRVECs) by activating the NLR family pyrin domain containing 3 (NLRP3). Both CCN1 and NOX4 are upregulated in DR (20, 24) and play important roles in the regulation of retinal inflammation and angiogenesis. However, whether CCN1 interacts with NOX4 in the pathogenesis of DR remains unknown.

In the current study, we reanalyzed the published transcriptome data of human retinal tissue (GSE102485) (25) to compare the mRNA levels of CCN1 and oxidative stress-related genes from DR patients and HCs. We also modulated CCN1 levels by overexpression or knocking-down to examine ROS generation, the expression levels of NOX4, and the junction molecule VE-cadherin in HRVECs. The current study demonstrated that CCN1 affects oxidative stress by modulating NOX4 and provides new insights into the mechanism of DR and a novel target for predicting or treating DR.

## Materials and Methods

### Ethics Statement

All experiments related to human samples were approved by the Clinical Research Ethics Committee of The Third Affiliated Hospital of Sun Yat-Sen University, according to the guidelines of the Declaration of Helsinki. All patients were recruited from the Department of Endocrinology at the same hospital. The C57BL/6J mice were purchased from Guangdong Medical Laboratory Animal Center (Guangzhou, Guangdong, China). All experiments were conducted according to the National Institute of Health's Guidelines for the Care and Use of Laboratory Animals, and approved by the Animal Research Ethics Committee of the Third Affiliated Hospital of Sun Yat-Sen University. All mice were housed under a standard 12-h light–dark cycle and specific pathogen-free conditions, and were allowed free access to food and water.

### Construction of Diabetic Mouse Model and Hyperlipidemic Mouse Model

The C57BL/6J mice, aged 6 weeks, were randomly divided into the diabetes mellitus (DM) group and normal control (NC) group. Mice were fed a high-fat diet and treated with streptozocin (STZ) to produce DM. The DM group was subjected to intraperitoneal injection of STZ at 40 mg/kg/day for 5 days, and the NC group was injected with a comparable amount of vehicle buffer. Blood glucose levels were measured daily before and after injection. Mice with random blood glucose levels above 16.7 mmol/L were considered to have DM. The ApoE^−/−^ mice, aged 6 weeks, were used for the hyperlipidemia model, and age-matched C57BL/6J mice were used as the control group.

### Sample Collection, RNA Extraction, and RNA Sequencing

We recruited three groups as follows: DR, DM control (DM without DR), and healthy controls (HCs). The main exclusion criteria for each group are shown in Supplementary Table 1. A volume of 6 ml of peripheral blood was collected, mixed with three times the volume of TRIzol, and stored in a refrigerator at −80°C for subsequent total RNA extraction. Furthermore, 3 μg RNA per sample was used as input for the RNA sample preparations. The RNA sequencing was performed by Novogene Inc. (Beijing, China).

### Database Mining

We searched the Gene Expression Omnibus (GEO) database that stores curated gene expression and original series and platform records in a repository associated with research studies about DR. We downloaded, from the GEO database, the accession number (GSE102485, https://www.ncbi.nlm.nih.gov/geo/query/acc.cgi?acc=GSE102485) (25) containing 19 neovascular membrane (NVM) samples from patients with proliferative diabetic retinopathy (PDR). Two PDR samples were not included, as the total number of sequencing reads were 10 times lower than the average. The FastQ files of our in-house RNA-seq data of peripheral blood from DR, DMC, and HC samples, as well as the downloaded RNA-seq data of NVM samples from patients with PDR (GSE102485) (25), were first mapped to the human genome (hg19) using the STAR tool. Gene expression was quantified using feature counts after normalization, based on the total number of mappable counts. Gene expression counts were further normalized among samples based on the total numbers of all mapped reads. Differentially expressed genes between any two groups (DMC vs. DR, HC vs. DR, etc.) were identified by using the cutoff values for fold enrichment at 2 and *p*-values of 0.05. Heatmaps and volcano plots were generated for the visualization of the deferentially expressed genes (DEGs). Principal component analysis (PCA) was also performed for the expression levels of all samples to illustrate the consistency of the replicates and possible similarities among the three groups. The PCA plots of the first principal components (PC1 and PC2) were used to interpret the potential sample similarity, only when the loss of information was below 25% in the two components. We further explored the functional annotation of theDGEs. The significantly enriched (FDR-q < 0.05) gene ontology (GO) terms were identified using the DAVID tools (https://david.ncifcrf.gov/). The Gene Set Enrichment Analysis (GSEA) tool (26) was further utilized to identify enriched gene sets (MSigDB 7.1) between any two groups of samples.

### Cell Culture

The HRVECs were obtained from Otwo Biotech (Shanghai, China) and cultured at 37°C with 5% CO_2_. Cells were maintained in Dulbecco's modified Eagle's medium (D6046; Merck, Darmstadt, Germany) containing 10% fetal bovine serum (SH30406.05: Hyclone, Logan, UT, USA) and 1% penicillin/streptomycin (SV30010-5; Hyclone). The HRVECs from passages 3–7 were used for further experiments.

### siRNA Treatment

Double-stranded small interfering RNA (siRNA) oligonucleotides against human NOX4 were synthesized by Obio Technology (Guangzhou, China) and a non-silencing siRNA was used as the negative control. Transfection was carried out using Lipofectamine 3000 (Invitrogen, Carlsbad, CA, USA) for 6 h. Cells were recovered after being left in normal culture medium overnight and were then treated with high concentrations of glucose or palmitate.

### CRISPR/Cas9-based CCN1 Knockout in HRVECs

The generation of CCN1-deficient HRVECs was performed as follows: Single guide RNA (sgRNA) oligos for human CCN1, 5′ GTTGTCATTGGTAACTCGTG, was used. The sgRNA oligo was inserted into lentiCRISPR v2 (Addgene plasmid 52961). We used second-generation lentiviral packaging plasmids (VSV-G and PAX2) to deliver CRISPR constructs into the cells. Twenty-four hours after infection, cells were cultured in a medium containing 2 μg/ml puromycin for 48 h, and then further cultured 24 h in normal growth medium.

### Antibodies

The anti-CCN1 antibody (26689-1) and anti-NOX4 antibody (14347) were purchased from Proteintech (Chicago, IL, USA). The anti-CD31 antibody (3528S) and tubulin (2128s) were purchased from Cell Signaling Technology (Cambridge, MA, USA). The anti-actin antibody (A5441) was purchased from Sigma-Aldrich (St. Louis, MO, USA). The anti-HSP90 antibody (ab13492), anti-ZO-1 antibody (ab59720), and anti-VE-cadherin antibody (ab33168) were purchased from Abcam (Cambridge, MA, USA). Isolectin GS-IB 4 from *Griffonia simplicifolia* and Alexa Fluor 488 conjugate (I21411) were purchased from Invitrogen (Carlsbad, CA, USA).

### Immunostaining Assays

The HRVECs were cultured on coverslips and fixed at the indicated time points with 4% paraformaldehyde in phosphate-buffered saline (PBS) for 15 min at room temperature. They were then subjected to 5 min of permeabilization with 0.3% Triton X-100. The coverslips were then blocked with 5% fetal calf serum, 1% bovine serum albumin, and 0.025% Tween 20 in PBS for 30 min at 37°C, followed by 4°C overnight incubation with primary antibodies. Subsequently, the Alexa Fluor conjugated secondary antibodies were applied for 1 h at room temperature and the nuclei were counterstained with 4′,6-diamidino-2-phenylindole (DAPI). The coverslips were then mounted onto glass slides and analyzed with fluorescence microscopy.

### Western Blot Analysis

The retinal tissue of mice and cell lysis was evaluated using western blot analysis. Both the homogenate of retinal tissues and HRVECs were extracted using radioimmunoprecipitation (RIPA) lysis buffer and placed on ice for 30 min. The lysates were centrifuged for 15 min at 12,000 × *g* at 4°C and the supernatants were collected. Protein concentration was determined using a bicinchoninic acid (BCA) assay kit. A 20-μg sample of each protein was separated by 10% sodium dodecyl sulfate-polyacrylamide gel electrophoresis at a specific voltage and then transferred onto a polyvinylidene difluoride (PVDF) membrane using a wet electro transferring system (Bio-Rad, Hercules, CA, USA). The PVDF membranes were incubated with a specific primary antibody overnight at 4°C, and then further incubated with appropriate secondary antibodies at room temperature for 1 h. The blots were visualized using an enhanced chemiluminescence (ECL) system (Bio-Rad, Hercules, CA, USA). The signal intensity of the target protein bands was quantitatively analyzed using the Image Lab software (Bio-Rad, Hercules, CA, USA) and normalized to the intensity of the internal control protein, such as actin or HSP90.

### Fluorescent Quantitative PCR

Total RNA was extracted from HRVECs using the TRIzol reagent (Invitrogen, Carlsbad, CA, USA). Quantitative real-time (RT)-PCR was conducted using the FastKing RT kit and SuperReal PreMix Plus kit (SYBR Green) (KR116-02 and FP205-02; Tiangen Biotech Co., Ltd., Beijing, China), according to the manufacturer's instructions. The GAPDH mRNA level was used as the internal control. The PCR program included one cycle at 95°C for 15 min; followed by 40 cycles at 95°C for 10 s; 60°C for 20 s; and 72°C for 30 s. Analysis of each sample was repeated three times. The primer sequences used are listed as follows:

GAPDH,

Forward 5′-GATGCTGGTGCTGAGTATGRCG-3′

Reverse 5′-GTGGTGCAGGATGCATTGCTCTGA-3′.

CCN1,

Forward 5′-GGTCAAAGTTACCGGGCAGT-3′

Reverse 5′-GGAGGCATCGAATCCCAGC-3′.

NOX4,

Forward 5′- CAGATGTTGGGGCTAGGATTG -3′

Reverse 5′- GAGTGTTCGGCACATGGGTA -3′.

VE-cadherin,

Forward 5′-TTGGAACCAGATGCACATTGAT-3′

Reverse 5′-GTGGTGCAGGATGCATTGCTCTGA-3′.

### Measurement of ROS Generation

The generation of ROS in HRVECs after palmitic acid (PA) treatment, or PA treatment together with CCN1 modulation, was determined using an ROS assay kit (Nanjing KeyGen Biotech Co. Ltd., Nanjing, Jiangsu, China) according to the manufacturer's instructions.

### Retina Flat-Mount and Immunostaining

Retinas were fixed in 4% paraformaldehyde (E672002-0500; Sangon Biotech, Shanghai, China) for 15 min at room temperature immediately after being resected from the mice, after which they were washed three times in PBS. Retinas were then flat-mounted, blocked, and permeabilized in blocking buffer for 1 h at room temperature. Isolectin GS-IB4 (I21411; Invitrogen, Carlsbad, CA, USA) was applied and retinas were left to incubate at 4°C overnight, followed by further incubation with anti-NLRP3 (cryo-2, AdipoGen) at 4°C overnight. The anti-mouse secondary antibody (ab150115, Abcam) was applied at room temperature for 1 h. After three washes with PBS, samples were mounted using the Vectashield HardSet mounting medium (H-1700-10; Vector Laboratories, Burlingame, CA, USA). The retinal vasculature was imaged using a confocal fluorescence microscope (TSC SP8; Leica Microsystems, Wetzlar, Germany). The density and lesions of the microvasculature were analyzed using the Image J software.

### Immunostaining of Frozen Section

The retinal frozen sections were blocked and permeabilized by a blocking buffer for 1 h at room temperature and further incubated with specific antibodies at 4°C overnight. After being washed three times with PBS, the sections were incubated with Alexa Fluor 488-conjugated anti-rabbit secondary antibody (4413; Cell Signaling) or Alexa Fluor 647 anti-mouse secondary antibody (ab150115; Abcam) at room temperature for 1 h. An inverted fluorescence microscope (Eclipse Ti-S; Nikon, Tokyo, Japan) was used for closer observation. The results were quantified using the Image-Pro Plus software (Media Cybernetics Inc., Rockville, MD, USA).

### Statistical Analyses

The results of continuous variables in the cohort study were presented as the mean ± SD. The baseline characteristics of each group were compared using the χ^2^ test or a one-way analysis of variance test. Statistical analyses were performed using the SPSS V.19.0 statistical software (SPSS). *p* < 0.05 was considered significant. To describe the biologically functional significance of selected gene sets, we used the hypergeometric test to determine the enrichment of GO terms and Kyoto Encyclopedia of Genes and Genomes (KEGG) pathways. The significance of differences was evaluated with either the Student's *t*-test when only two groups were compared, or the hypergeometric test for a Venn diagram. Hierarchical clustering was performed using the Cluster 3.0 software.

## Results

### Distinct Patterns Are Observed in the Transcriptome of Retinal Tissues and Peripheral Blood Mononuclear Cells From Patients With DR

Because of limited sample resources, only a few studies have demonstrated the RNA expression profiles of retinal samples from patients with DR. One such study, GSE102485 (25), analyzed three retinal samples from healthy controls (HCs), two retinal samples from patients with type 2 diabetes, and 25 retinal neovascular membranes (NVMs). Among the NVM samples, three were from patients with branch retinal vein occlusion, three from those with type 1 diabetes, two from those with retinal periphlebitis, and 17 from those with type 2 diabetes and PDR. We reanalyzed the raw data from that study and included 17 samples that fit our purpose (Supplementary Figures 1A–C). Therefore, we included 12 retinal NVM samples, three normal samples, and two type 2 diabetes samples.

**Figure 1:**
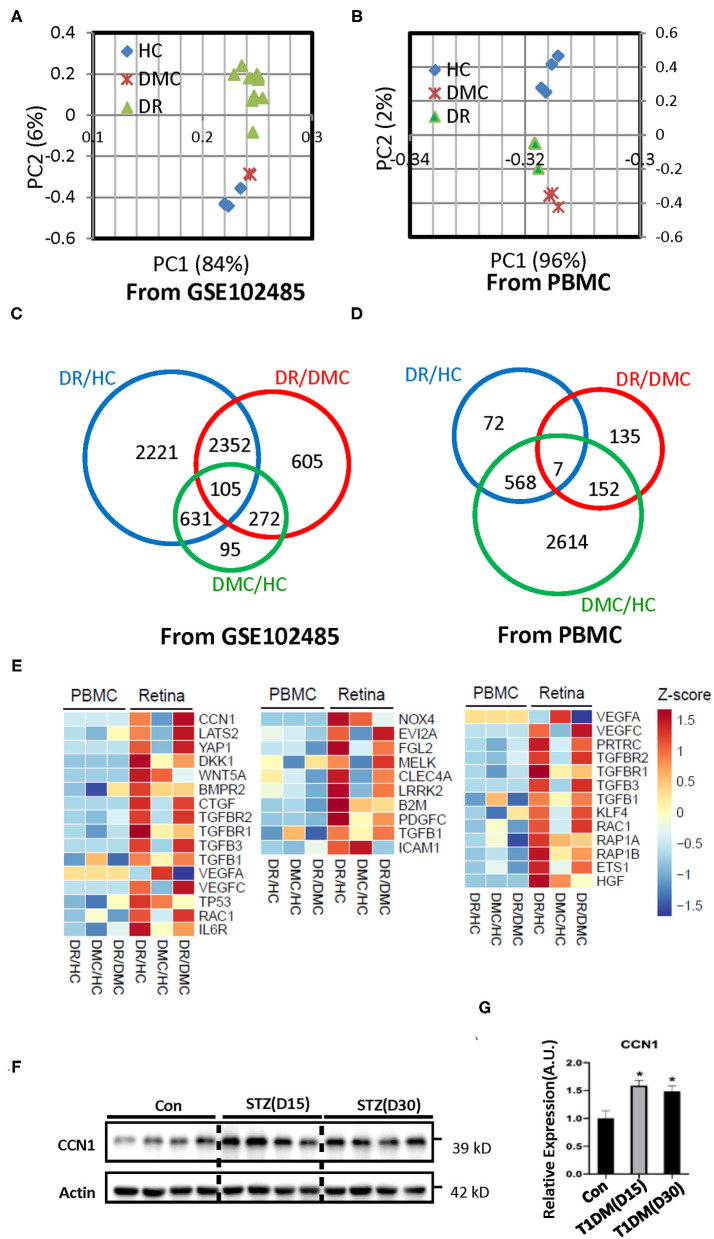
Transcriptome profiles of retina tissue (the raw data of GSE102485) and peripheral blood samples from healthy controls (HCs), the diabetes mellitus control (DMC), and patients with diabetic retinopathy (DR). **(A,B)** Principal component analysis of the sequenced samples revealed the separation between DR samples and the other samples. **(C,D)** Venn diagrams summarize the different expression profiles among the DR, DMC, and HC groups. **(E)** The heat maps show different transcriptome profiles between retinal tissue and peripheral blood mononuclear cells (PBMCs); the image on the left represents the gene group associated with CCN1, the image in the middle represents the gene group associated with oxidative stress, and the image on the right represents the gene group associated with VE-cadherin. **(F)** Western blot analysis and **(G)** densitometry quantification of CCN1 in retinas from streptozotocin (STZ)-induced hyperglycemic mice. *p < 0.05.

The PCA of the transcriptomes showed congruent expression profiles among the three groups, whereas the similarity between the DM group and the HC group was greater (Figure 1A). In the peripheral blood samples, the PCA characteristics of the DR and DM groups showed greater similarity (Figure 1B). The Venn diagram constructed identified 2,221 differentially expressed genes between the NVMs of patients with DR and the retinal tissues of HCs. In addition, 95 differentially expressed genes were identified between retinal tissues of patients with DM and those of HCs (Figure 1C). Those genes were in the main categories related to extracellular matrix (ECM)–receptor interaction, tight junctions, apoptosis, and metabolism, among other categories (Supplementary Figure 2A). Although 72 differential gene expressions were identified in the peripheral blood of patients with DR and HCs, 2,614 gene differences were identified in the peripheral blood of patients with DM and HCs (Figure 1D). The genes involved in the GO category related to tight junctions were also more involved with inflammation or immune-related categories (Supplementary Figure 2B).

The transcriptome profiles of the retinal tissue are related to the pathophysiology of DR. The whole body is connected by the circulatory system and the blood is an accessible sample to show the distinct biomarker profile of heart failure with type 2 diabetes mellitus (27). Thus, we sought to determine whether the features of the DR transcriptome profile can be reflected in the peripheral blood sample. We enrolled three patients with DR, three patients with DM, and four HCs. The peripheral blood samples were collected and total RNA was extracted. The transcriptome profiles were obtained through the deep sequencing technique. The sequence quality was good enough for subsequent analysis (Supplementary Figure 1B). To discriminate between expression profiles between the retinal tissue and peripheral blood samples, we constructed a heat map of genes associated with DR-related pathways (Figure 1E; Supplementary Figures 3A–D). The transcriptome characteristics of retinal tissue cannot be reflected in peripheral blood.

As one of our aims was to illustrate the pathogenesis of DR, we chose to focus on the results of the retinal tissue. The cap junction molecular, oxidative stress, and the endoplasmic reticulum membrane protein complex (EMC) molecular pathways are considered to be related to the pathogenesis of DR. The heat map indicated the absolutely differential expression levels of genes associated with DR-related pathways. The expression of most of the VE-cadherin-, NOX4-, and CCN1-related genes was increased in the retina of patients with DR. VE-cadherin-related genes upregulated the body's ability to compensate for diseases. The EMC molecule, CCN1, is positively associated with DR, but its underlying mechanism is still unclear. Our results suggest that CCN1 influenced the tight junction through NOX4-induced oxidative stress. Interestingly, our results showed that the expression level of CCN1 in the retina of mice with diabetes was significantly higher than that of control mice (Figures 1F,G).

### CCN1 Is Upregulated in Retinal Vessels From Mice With Hyperglycemia or Hyperlipidemia

**Figure 2:**
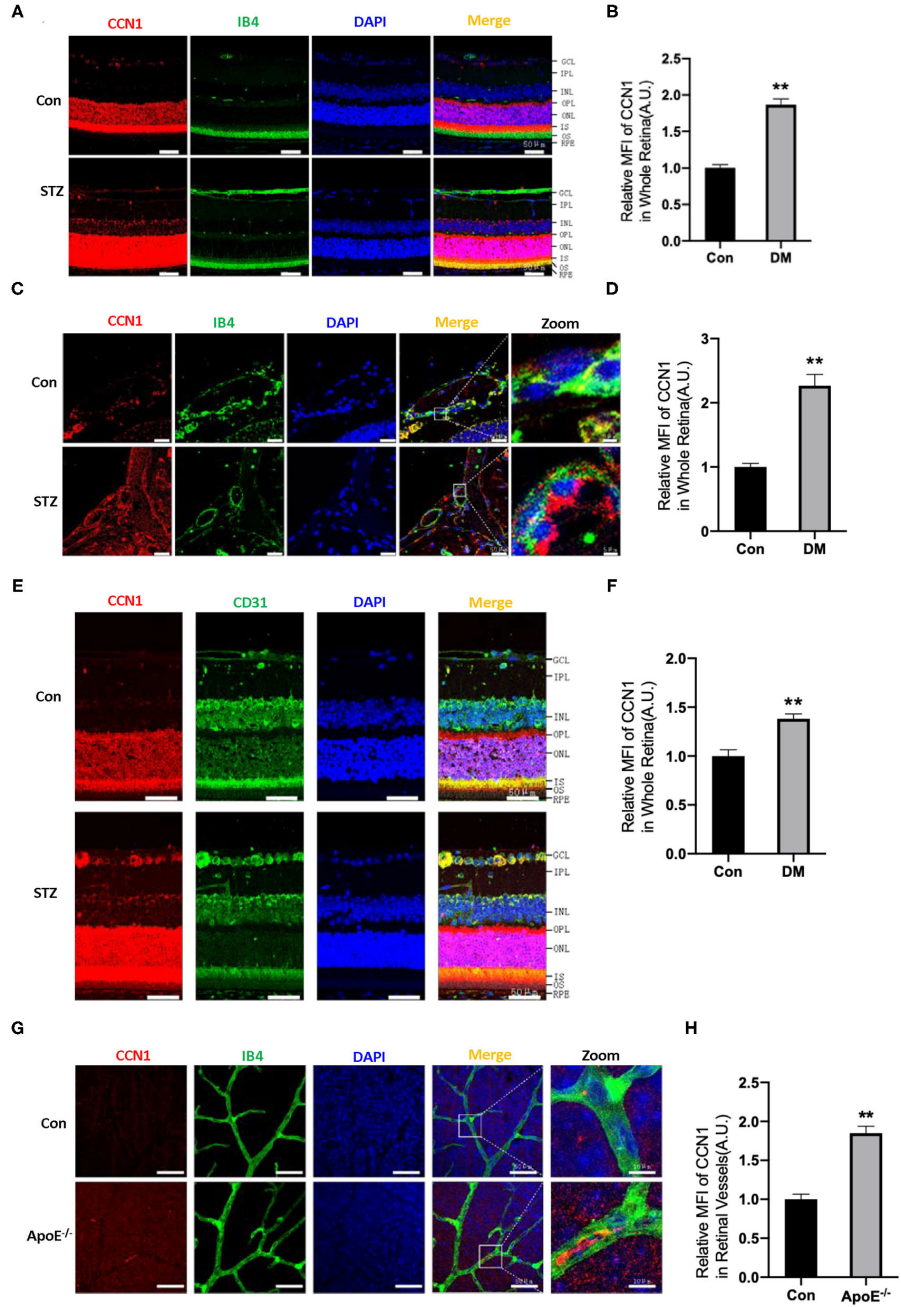
CCN1 expression is increased in the retinal vessels of mice with diabetes mellitus (DM) and ApoE^−/−^ mice. **(A)** Representative images of CCN1 expression patterns and **(B)** quantification of CCN1 mean fluorescence on frozen sections of the retina from control mice and streptozotocin (STZ)-induced hyperglycemic mice on day 15 after modeling; Isolectin B4 (IB4) was the vascular marker. **(C)** Representative images and **(D)** quantification of CCN1 mean fluorescence of central vessels on frozen sections of the retina from control mice and STZ-induced hyperglycemic mice on day 15 after modeling. **(E)** Representative images of CCN1 expression patterns and **(F)** quantification of CCN1 mean fluorescence on frozen sections of the retina from control mice and STZ-induced hyperglycemic mice on day 15 day after modeling; CD31 was the vascular marker. **(G)** Representative images of CCN1 expression pattern and **(H)** quantification of CCN1 mean fluorescence on flat-mount sections of the retina of ApoE^−/−^ mice and control mice; IB4 was the vascular marker. **(A,C,E,F)** Scale bar = 50 μm. **p < 0.01.

The ECM is important for maintaining the normal function of blood vessels. Although CCN1 is an ECM component, closely related to DR, its role in the pathogenesis of DR is unclear. In order to determine whether CCN1 is involved in the pathogenesis of DR, we established a DM mouse model through STZ injection. Mice with blood glucose levels higher than 16.7 mmol/L were considered diabetic. Metabolic disorders, especially hyperlipidemia, are another characteristic of patients with DM. Therefore, ApoE^−/−^ mice were used to study the effects of hyperlipidemia on the expression of CCN1 in retinal vascular tissue. *In situ* immunofluorescence experiments indicated that, compared with control mice, the expression of CCN1 in the whole retinal tissue of mice with DM was significantly increased (Figures 2A,B,E,F). To locate retinal blood vessels, we used IB4 and CD31 as vascular markers. We found the expression of CCN1 to be significantly increased in the retinal vascular tissue of diabetic mice using either marker. We also detected and analyzed the expression of CCN1 in the central artery and vein. As expected, CCN1 expression around the IB4-marked vessels was significantly increased in diabetic mice (Figures 2C,D). As both hyperglycemia and hyperlipidemia are the main metabolic features of DM, we investigated how hyperlipidemia affects CCN1 expression in ApoE^−/−^ mice. We observed increased CCN1 expression in ApoE^−/−^ mice (Figures 2G,H).

### CCN1 Expression Is Induced by Palmitic Acid or High Glucose Treatment in HRVECs

**Figure 3:**
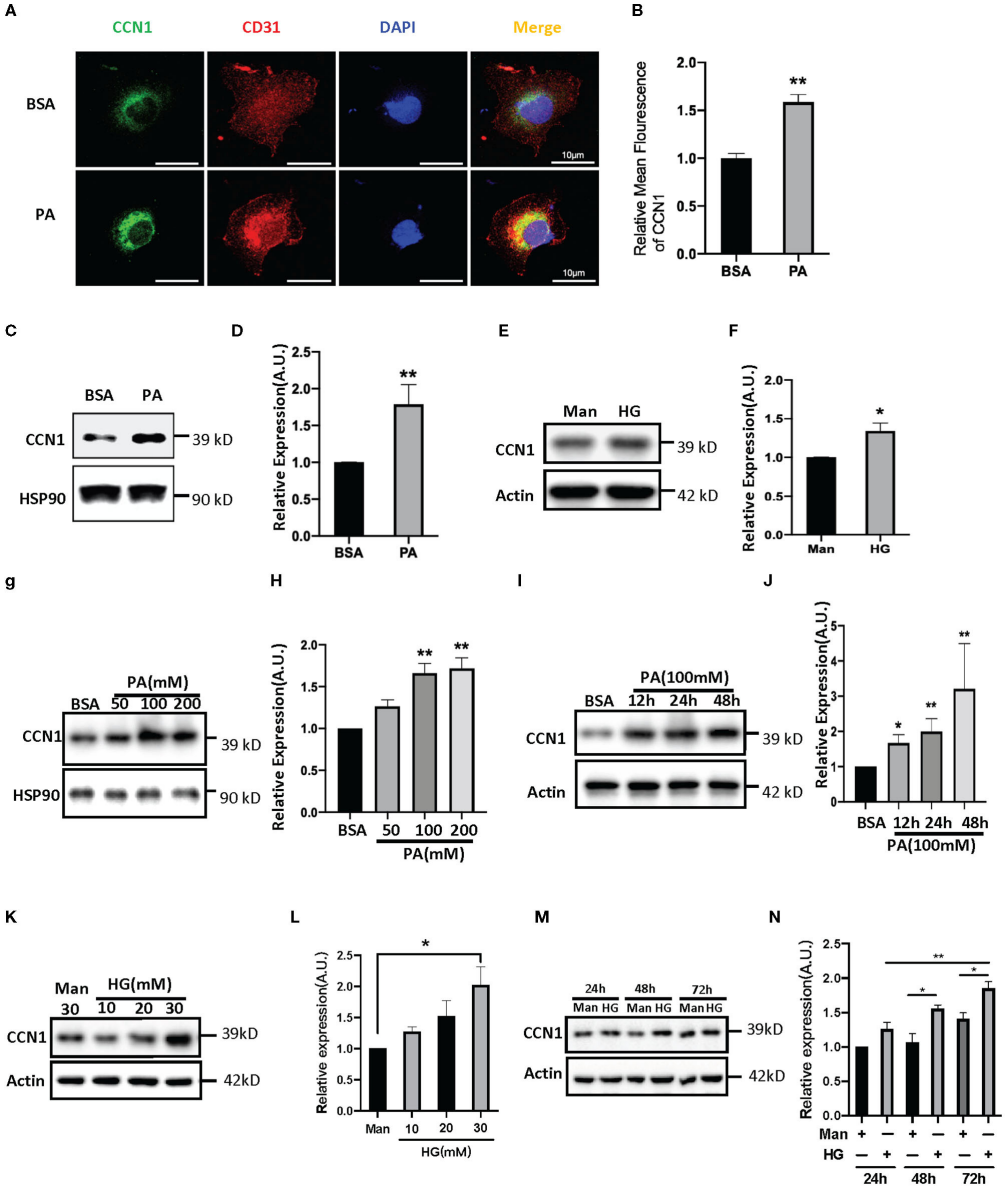
Palmitic acid (PA) and high glucose (HG) levels induce CCN1expression in human retinal vascular endothelial cells (HRVECs). **(A)** Representative images and **(B)** quantification of CCN1 mean fluorescence in HRVECs treated with 100 mM PA for 24 h, scale bar = 10 μm. **(C,E)** Western blot analysis and **(D,F)** densitometry quantification of CCN1 in HRVECs under 100 mM PA or 30 mM HG treatment, respectively. **(G)** Western blot analysis and **(H)** densitometry quantification of CCN1 in HRVECs under 50, 100, or 200 mM PA for 24 h. **(I)** Western blot analysis and **(J)** densitometry quantification of CCN1 in HRVECs under 100 mM PA treatment for 12, 24, and 48 h. **(K)** Western blot analysis and **(L)** densitometry quantification of CCN1 in HRVECs under 10, 20, or 30 mM HG for 48 h. **(M)** Western blot analysis and **(N)** densitometry quantification of CCN1 in HRVECs under 30 mM HG treatment for 24, 48, and 72 h. *p < 0.05, **p < 0.01.

Both lipotoxicity and glucotoxicity play important roles in promoting the progression of diabetic microvascular complications; however, which play a more important role is not clear. Hyperglycemia is considered the driving cause of DR pathology (28), while several studies found DR pathology in patients with relatively normal glucose tolerance and suggest that glucose may not be the primary driver of DR (29, 30). Recent studies indicate a strong association between dyslipidemia and DR (31, 32). In order to explore the influence of the DM environment on CCN1 expression in retinal microvascular cells, we chose HRVECs as the cell model. After 24 h of treatment with palmitic acid, the immune-stained HRVECs showed that PA can significantly increase the levels of CCN1, and most of the CCN1 can be found around the nucleus (Figures 3A,B). Western blot analysis also confirmed that CCN1 levels were upregulated after both PA (100 mM, Figures 3C,D) and high glucose (HG 30 mM, Figures 3E,F) treatment over 24 h; however, the difference was not as significant as that of the PA-treated cells. We then treated HRVECs with different concentrations of PA and HG for different durations. The Western blot analysis showed that PA treatment over 24 h dose-dependently induced both the protein levels of CCN1 in HRVECs (Figures 3G,H). We also treated HRVECs with PA (100 mM) for different durations (Figures 3I,J) and optimized the PA stimulation conditions. The results of Western blot analysis showed that HRVECs treated with PA at a concentration of 100 mM for 24 h were the best conditions. We also treated HRVECs with different concentrations of HG for different durations and the effect on the CCN1 expression is not significant than PA (Figures 3K–N).

### CCN1 Inhibits VE-cadherin Expression in HRVECs

**Figure 4:**
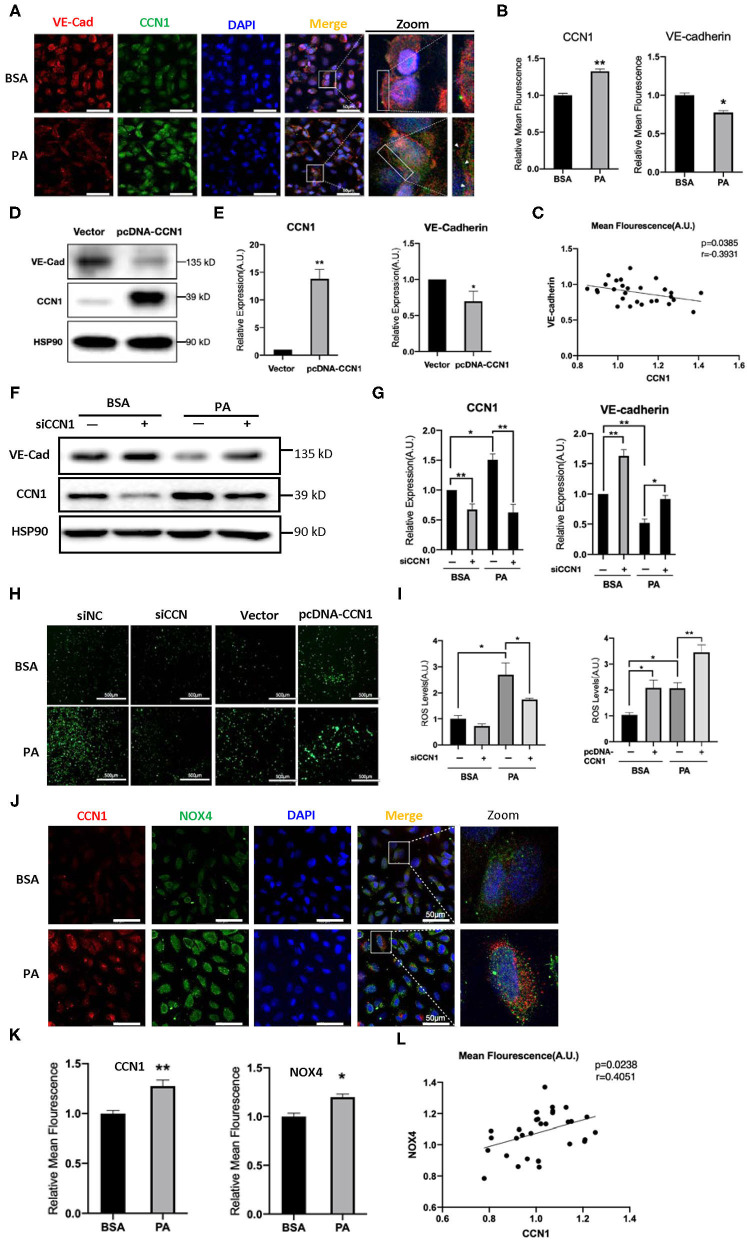
CCN1 downregulates VE-cadherin by activating the NADPH oxidase (NOX4)/reactive oxygen species (ROS) axis. **(A)** Representative images, **(B)** mean density, and **(C)** relevance analysis of fluorescence of CCN1 and VE-cadherin in human retinal vascular endothelial cells (HRVECs). **(A)** White arrows indicate the defect of VE-cadherin on the cell membrane, scale bar = 50 μm. **(D)** Western blot analysis and **(E)** densitometry quantification of VE-cadherin and CCN1 in HRVECs after transfection with CCN1 plasmids or vector. **(F)** Western blot analysis and **(G)** densitometry quantification of VE-cadherin and CCN1 in HRVECs transfected with siCCN1 or the negative control, followed by 100 mM PA or bovine serum albumin (BSA) treatment for 24 h. **(H)** Representative images and **(I)** measurement of ROS generation in HRVECs transfected with siCCN1 or the negative control (siNC) followed by 100 mM PA or BSA treatment for 24 h, scale bar = 500 μm. **(J)** Representative images, **(K)** mean density, and **(L)** relevance analysis of fluorescence of CCN1 and NOX4 in HRVECs, scale bar = 50 μm. *p < 0.05, **p < 0.01.

The results above indicated that lipotoxicity in DM can stimulate the expression of CCN1, but it is unclear whether increased CCN1 affects the function of retinal vascular endothelial cells. VE-cadherin is an endothelium-specific member of the tight junctions, and its reduction is related to the leakage of the retinal vasculature (33). We performed immunofluorescence staining to observe the expression patterns of VE-cadherin and CCN1 in HRVECs treated with 100 mM PA for 24 h. We found that PA can increase the expression of CCN1 and reduce the expression of VE-cadherin, which indicates that the levels of CCN1 and VE-cadherin are negatively correlated (Figures 4A–C).

To determine whether CCN1 actually regulates the expression of VE-cadherin, we induced the overexpression of CCN1 in HRVECs using plasmids. The vacant adenovirus vector was used as a control (vector group). Compared with the control group, the expression of CCN1 in the pcDNA-CCN1 group was significantly increased, whereas the expression of VE-cadherin was reduced (Figures 4D,E). Furthermore, we downregulated the expression of CCN1 using small interfering RNA (siCCN1). Both siCCN1-1 and siCCN1-2 can effectively inhibit the expression of CCN1 (Supplementary Figure 5). Consistent with the previous results (Figure 3C), PA treatment upregulated CCN1 expression in HRVECs, but downregulated VE-cadherin expression. In addition, knockdown of CCN1 significantly upregulated the expression of VE-cadherin, compared with the scramble control group (Figures 4F,G). Therefore, CCN1 can directly regulate VE-cadherin expression in HRVECs.

Moreover, CCN1 reportedly stimulates ROS generation (34) and ROS can potently downregulate the expression of VE-cadherin (35). As there is a clear relationship between ROS overproduction and DR (36), we proposed that ROS may mediate the inhibitory effect of CCN1 on VE-cadherin in HRVECs. Therefore, we measured ROS levels in the siCCN1 and pcDNA-CCN1 groups. Our results showed that PA treatment significantly increased the ROS levels, whereas siCCN1 attenuated ROS induction, and pcDNA-CCN1 potentiated ROS induction (Figures 4H,I). These findings indicate that the inhibitory effect of CCN1 on the expression of VE-cadherin is mediated through ROS production. As NOX4, which produces ROS, is abundantly expressed in vascular endothelial cells (37) and reportedly causes the destruction of the blood-brain barrier (22), we examined the levels of CCN1 and NOX4 in HRVECs after PA treatment. Immunostaining findings showed that levels of both CCN1 and NOX4 were increased in PA-treated HRVECs (Figures 4J–L), suggesting a positive correlation between the expression levels of CCN1 and NOX4. These results indicate that CCN1 may inhibit the expression of VE-cadherin by activating the NOX4/ROS axis.

### CCN1 Inhibits VE-cadherin Expression by Upregulating NOX4 in HRVECs

**Figure 5:**
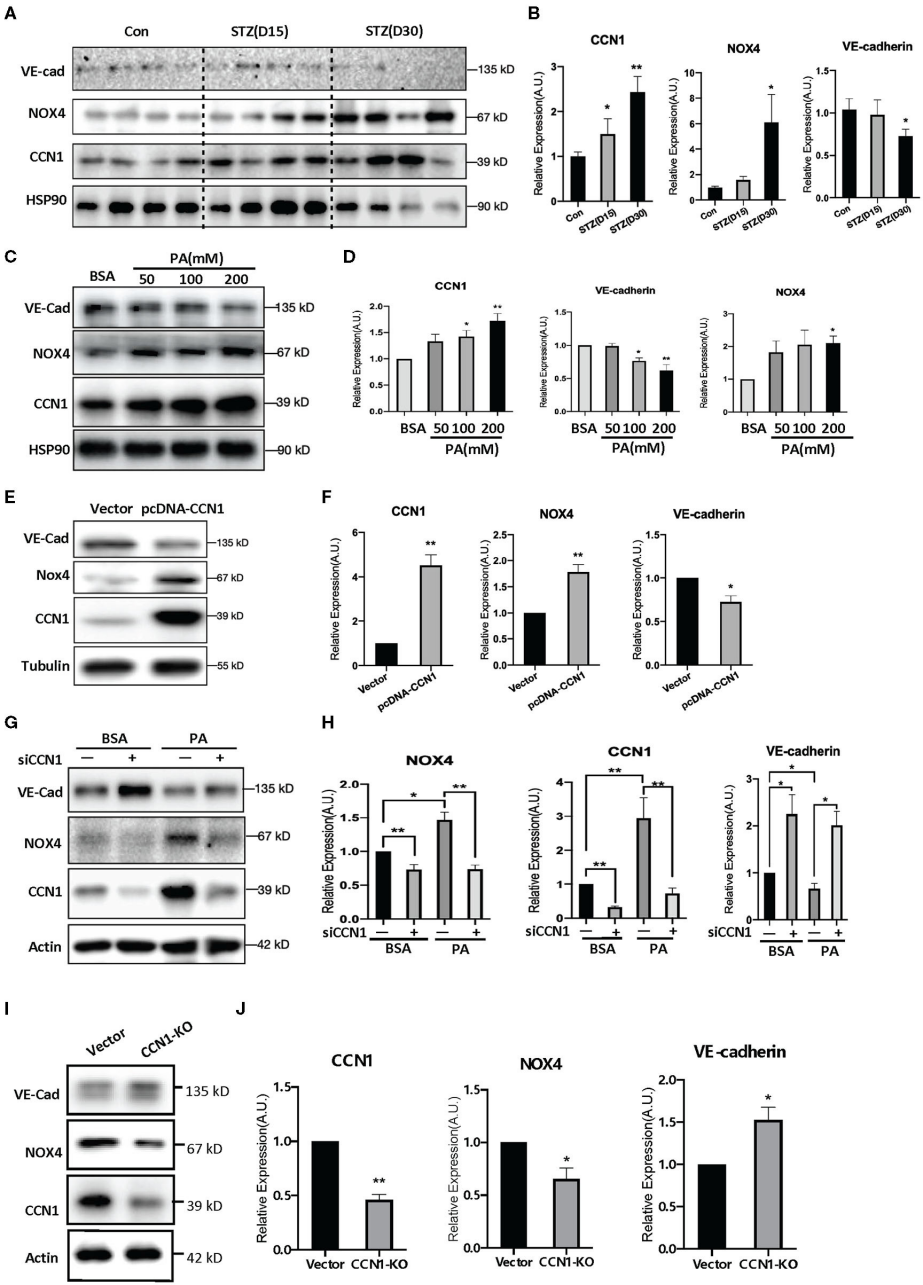
NADPH oxidase (NOX4) mediates the negative regulatory effects of CCN1 on VE-cadherin expression. **(A,C,E,G,I)** Western blot analysis and **(B,D,F,H,J)** densitometry quantification of protein expression levels of VE-cadherin, NOX4, and CCN1 in **(A,B)** retina tissue of control mice and streptozotocin (STZ)-induced hyperglycemic mice on days 15 and 30 after modeling; **(C,D)** human retinal vascular endothelial cells (HRVECs) treated with serial dosages (50, 100, or 200 mM) of palmitic acid (PA) for 24 h; **(E,F)** HRVECs transfected with CCN1 plasmids or vector; **(G,H)** HRVECs transfected with siCCN1 or the negative control (siNC), followed by treatment with 100 mM PA or BSA for 24 h; **(I,J)** CCN1-KO HRVECs via CRISPR. *p < 0.05, **p < 0.01.

Oxidative stress is one of the pathogenic factors leading to DR (38). Our findings above indicate a positive correlation between the levels of CCN1 and NOX4. To further determine whether NOX4 mediates the inhibition of VE-cadherin expression by CCN1, we tested the levels of CCN1, NOX4, and VE-cadherin in the retinas of mice with STZ-induced DM. Western blot analysis showed that, compared with the control group, CCN1 and NOX4 were significantly increased in the retinas of mice after 30 days of initial STZ treatment, whereas VE-cadherin was significantly reduced (Figures 5A,B). A similar expression pattern was also observed in PA-treated HRVECs (Figures 5C,D).

Moreover, we tested the expression levels of NOX4 and VE-cadherin after CCN1 overexpression. Our results showed that the expression of NOX4 was significantly increased in the pcDNA-CCN1 group compared with the vector group (Figures 5E,F). Furthermore, we measured the expression levels of NOX4 and VE-cadherin after CCN1 downregulation using siCCN1. In contrast to the effects of overexpression, NOX4 was significantly downregulated, whereas VE-cadherin was upregulated (Figures 5G,H). In addition, we constructed the CCN1 knockout (CCN1-KO) HRVECs using CRISPR-Cas9 plasmids. The target sequence and efficiency map of CCN KO is shown in Supplementary Figure 4. Consistently, similar effects to those of siCCN1 were detected in CCN1-KO HRVECs (Figures 5I,J). These results confirm that CCN1 negatively regulates the expression of VE-cadherin and positively regulates the expression of NOX4.

**Figure 6:**
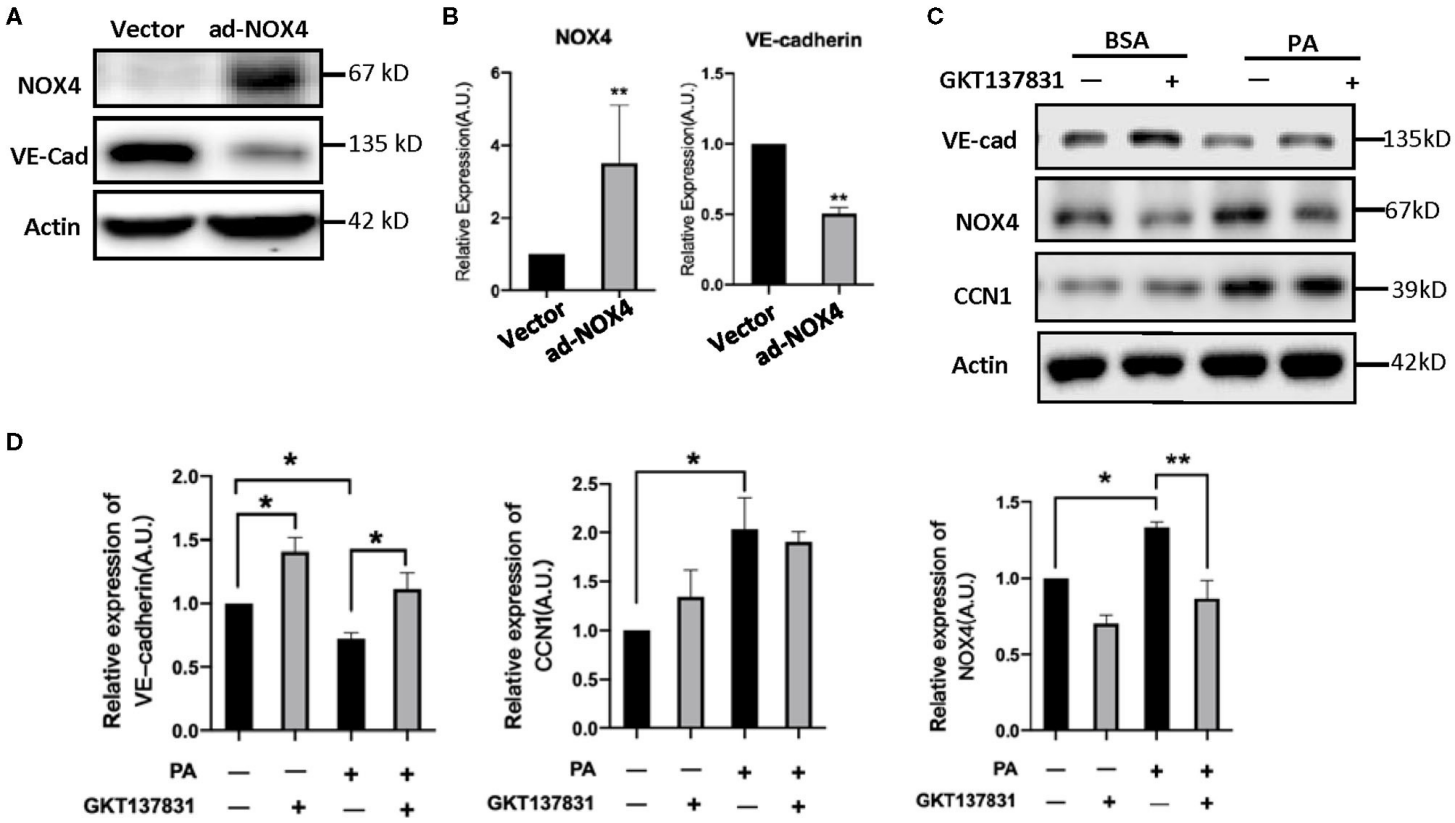
CCN1 positive regulate the expression of NOX4 in HRVECs. **(A,B)** HRVECs infected with NOX4-overexpressed adenovirus or vector; **(C,D)** The active of NOX4 was inhibited by GKT137831 in HRVECs. *p < 0.05, **p < 0.01.

In order to determine the NOX4 regulation of VE-cadherin expression, we modulated the levels of NOX4 in HRVECs using ad-NOX4. In order to clarify the regulation relationship between NOX4 and VE-Cadherin, we treat the HRVECs with the NOX4 inhibitor GKT137831. Western blot analysis showed that the overexpression of NOX4 can significantly reduce the levels of VE-cadherin (Figures 6A,B), while GKT137831 increases the level of VE-cadherin (Figures 6C,D) indicating that NOX4 negatively regulates the expression of VE-cadherin.

## Discussion

Although DR is the leading cause of blindness in developed and developing countries (39), the underlying pathogenic mechanisms have not been fully elucidated. The CCN family proteins are composed of four domains (IGFBP, VWC, TSP1, and CT) and functions at the cell matrix boundary or within the nucleus to modulatory gene transcription (40). The first identified member of the CCN family was CCN1, which was described as an angiogenic factor (41). The functionality of retinal small vessels depends on the subendothelial matrix, which is rich in CCN1 (42, 43). Previous reports have shown that CCN1 expression is increased in the retinas from mice with DM and from patients with DR, as well as in the vitreous humor of patients with PDR (15, 16, 44). Furthermore, circulating CCN1 levels are significantly correlated with the severity of peripheral arterial disease in diabetic patients (45). Other studies have shown that CCN1 regulates VEGF signaling during DR progression (17, 45). Those studies suggest a possible role of CCN1 in the pathogenesis of DR. However, the detailed mechanism underlying the involvement of CCN1 in DR pathogenesis remained unclear.

**Figure 7:**
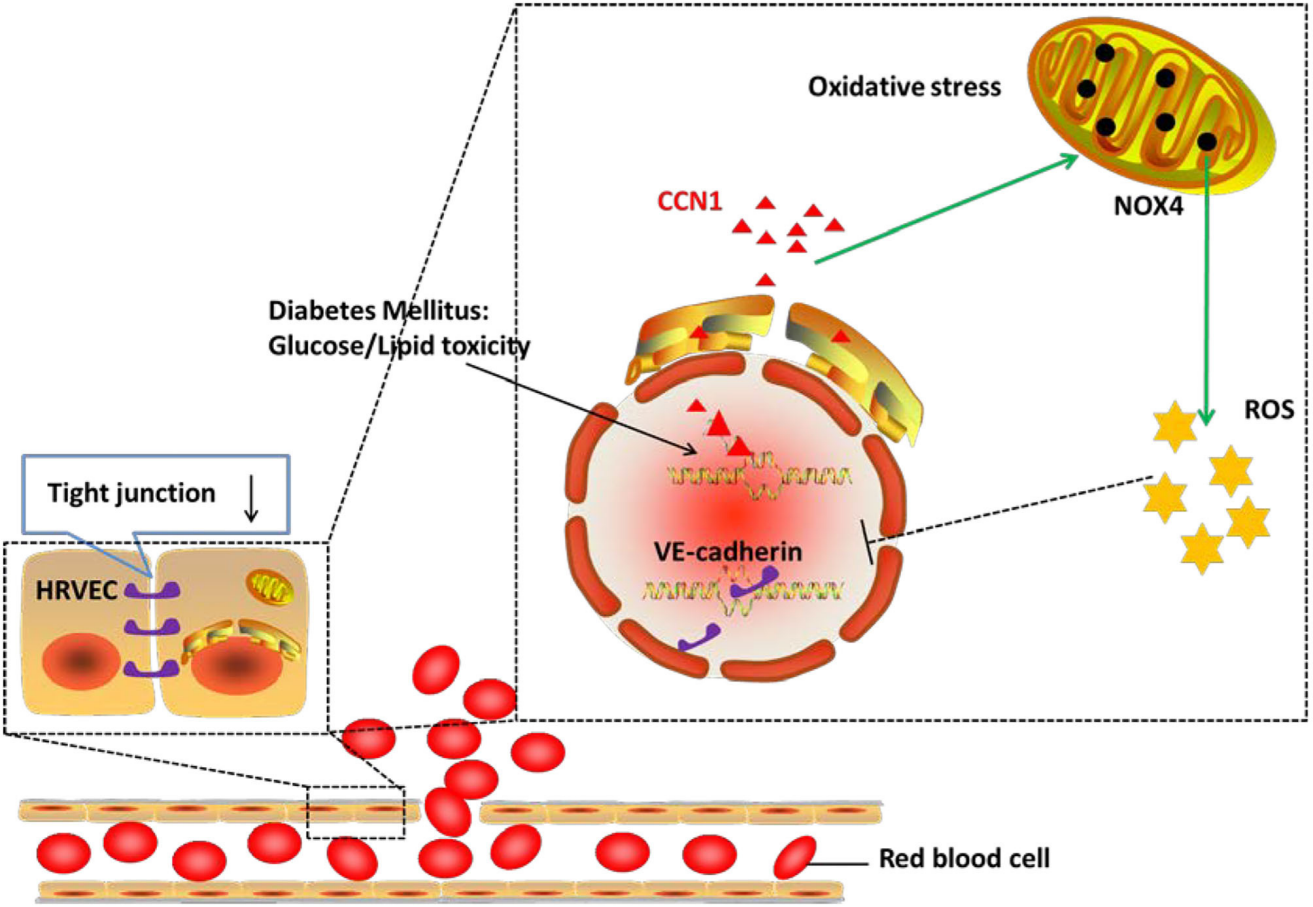
Mechanism of action of CCN1 in the pathogenesis of diabetic retinopathy (DR). The abnormal increase in CCN1 results from either the lipotoxicity or glucotoxicity of diabetes mellitus and leads to upregulated NOX4/ROS production, which further inhibits the expression of the tight junction protein.

In the current study, we demonstrated that the expression of CCN1 was significantly upregulated in the microvascular endothelial cells from DM patients, as well as in diabetic mouse models. We further confirmed that CCN1 expression could be induced by either high glucose or fatty acid, while CCN1 induction inhibited tight junction protein expression by activating the NOX4/ROS axis. Overall, as summarized in Figure 7 our results revealed a previously unappreciated mechanism of CCN1 regulation and function in promoting oxidative stress and tight junction damage in microvascular endothelial cells, which also suggested a potential strategy for the treatment of DR by targeting CCN1.

Previous reports have shown that ROS overproduction induced by hyperglycemia or hyperlipidemia plays key roles in endothelial damage in patients with DM (46). NOX4 is the main enzyme generating ROS in endothelial cells, and CCN1 induction leads to an accumulation of ROS (21). We compared the RNA expression profiles between retinal tissues and PBMCs from DR patients and non-diabetic controls. We noticed that the mRNA expression profile of endothelial cells from the retinas of DR patients (GSE102485) (25) showed increased levels of CCN1and oxygen stress-related genes compared with non-diabetic controls. These findings were further confirmed in the retina tissues from mice with STZ-induced diabetes. Furthermore, we demonstrated that both high glucose levels and PA treatment induced the expression of CCN1 in HRVECs *in vitro*. These results are consistent with previous studies in that both the volume of NOX4/ROS and the expression level of CCN1 are upregulated under DM. Our results also confirm that DM status can promote NOX4/ROS production through CCN1 upregulation.

Damage of the blood-retinal barrier (BRB) is an early feature of DR that results in vascular leakage, as well as macular edema, which can cause distortion and loss of central vision (17). The proteolytic degradation of VE-cadherin plays an important role in the breakdown of the BRB and increases retinal vascular permeability (33). The interactions between the endothelium and ECM are important for maintaining the normal functions of blood vessels. A previous study has shown that NOX4-generated ROS induces neuronal and blood-brain barrier injury after intracerebral hemorrhage (22). Besides, AGE treatment promoted NOX4 membrane translocation, ROS production, and VE-cadherin phosphorylation and degradation in human umbilical vein endothelial cells (47). Acarbose treatment in diabetic rats blocked NOX4-dependent superoxide generation in rat aortic endothelial cells and ameliorated vascular leakage by upregulating VE-cadherin expression (48). Moreover, CCN1 is critical for retinal vascular development (49). Through the analysis of public database, clinical samples, mouse models, as well as HRVECs *in vitro*, the current study demonstrates that CCN1 is induced in vascular endothelial cells under diabetic conditions, and that CCN1 disrupts the intercellular tight junction of the retinal vascular endothelium via NOX4 induction and ROS overproduction to negatively regulate VE-cadherin protein level. However, the mechanism on how CCN1 is regulated by hyperglycemia or hyperlipidemia and how CCN1 regulates NOX4 expression remains unclear. Further, whether CCN1 regulates VE-cadherin by inducing NOX4 *in vivo* and whether inhibiting CCN1 expression will protect against DR progression in animal models remain to be explored.

In summary, we demonstrated that up-regulating CCN1 expression under DM activated the NOX4/ROS axis in microvascular endothelial cells and led to vascular leakage through inhibiting VE-cadherin expression. The current study has identified a novel and important role of CCN1 in the pathogenesis of DR and may thus provide a potential target for DR therapy through inhibiting CCN1 expression or signaling.

## Data Availability Statement

The datasets presented in this study can be found in online repositories. The names of the repository/repositories and accession number(s) can be found below: SRA, PRJNA722765.

## Ethics Statement

The studies involving human participants were reviewed and approved by Clinical Research Ethics Committee of The Third Affiliated Hospital of Sun Yat-sen University. The patients/participants provided their written informed consent to participate in this study. The animal study was reviewed and approved by Animal Research Ethics Committee of the Third Affiliated Hospital of Sun Yat-Sen University.

## Author Contributions

HL wrote the original version of the manuscript. HL, TL, and HW performed most of the experiments. YLi and HY helped with the bioinformatic analysis of the GSE102485 and the PBMC RNA-seq data. YLu, SW, RP, and YN provided technical assistance. XH helped to organize and write the manuscript and performed some of the experiments. TL and HW helped to write the manuscript. YC, GS, and YY initiated and designed the study and experiments and edited the manuscript. All authors contributed to the article and approved the submitted version.

## Conflict of Interest

The authors declare that the research was conducted in the absence of any commercial or financial relationships that could be construed as a potential conflict of interest.

## Publisher's Note

All claims expressed in this article are solely those of the authors and do not necessarily represent those of their affiliated organizations, or those of the publisher, the editors and the reviewers. Any product that may be evaluated in this article, or claim that may be made by its manufacturer, is not guaranteed or endorsed by the publisher.
