# Supplementary material for: Diabetes Promotes Retinal Vascular Endothelial Cell Injury by Inducing CCN1 Expression
Source: Front Cardiovasc Med. 2021 Aug 11;8:689318. doi: 10.3389/fcvm.2021.689318 (PMC8385274; doi:10.3389/fcvm.2021.689318)
Supplement: Supplementary file 1 [file Data_Sheet_1.PDF]

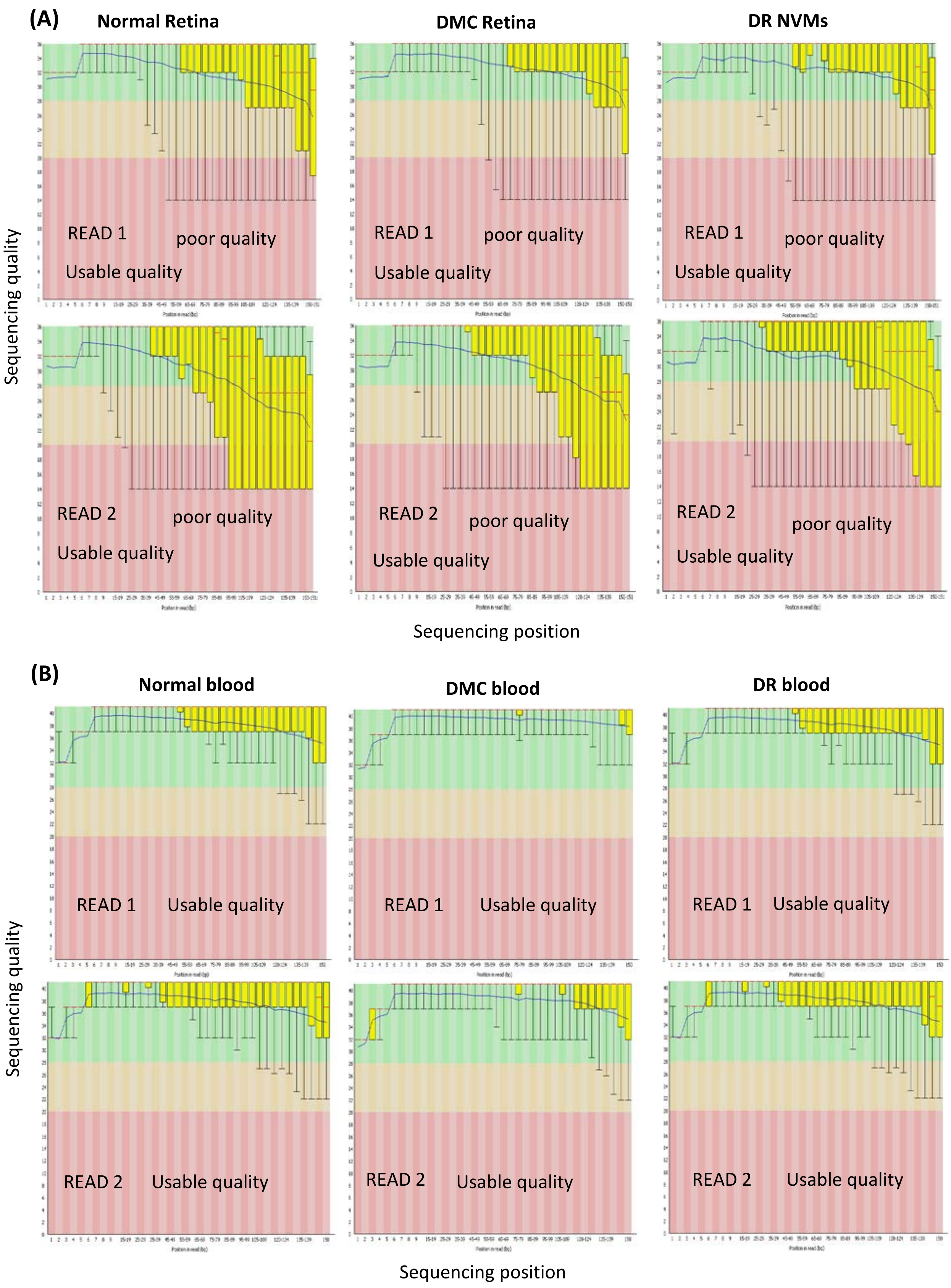

Figure S1. Sequencing quality of Retina from literature GSE102485 and PBMC. (A) the quality control result of the GSE102485 RNA-sequencing data; (B) the quality control result of the PBMC RNA-sequencing data.

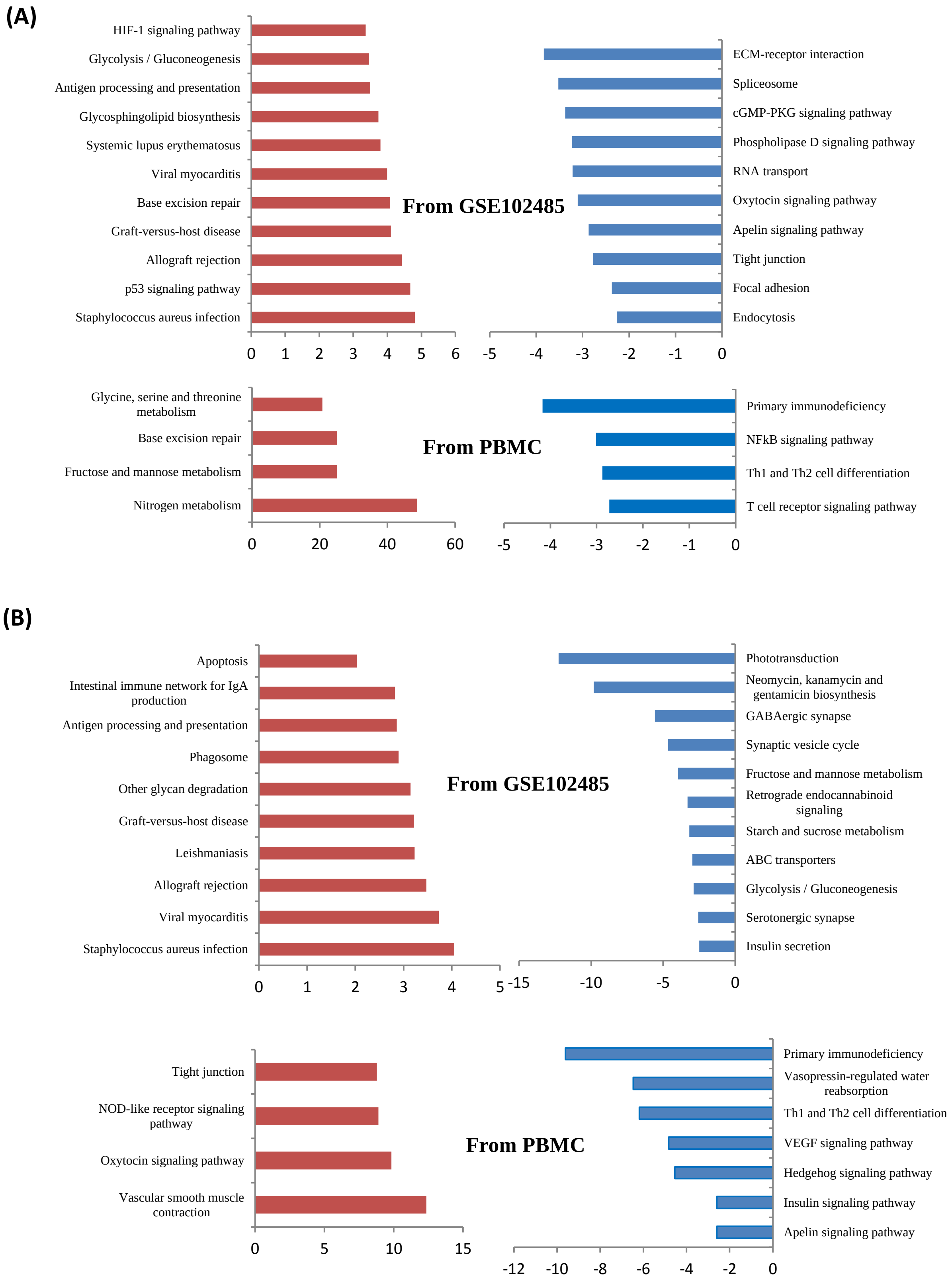

Figure S2. The Result of Genes Enrichment Analysis The top pathways are presented in order of descending significance with the number of genes from the down regulated (blue color) or up regulated (red color) genes in DMC group compared with the HC group list enriching the pathway **(A)** and in DR group compared with the HC group **(B)**.

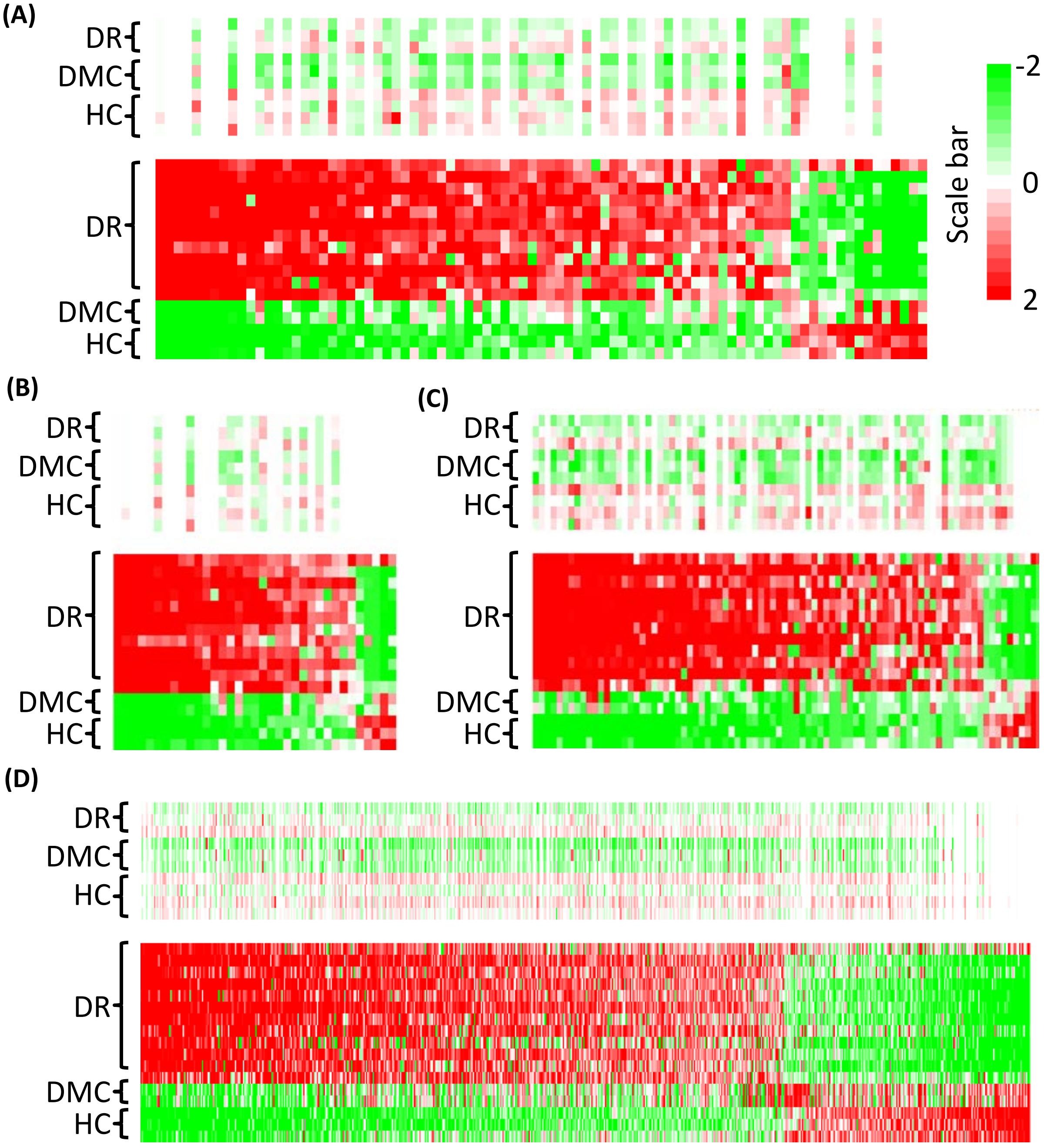

Figure S3. Heat maps show differential transcriptome profiles of four relevant pathways for both PBMC (upper panel) and tissue (bottom panel，GSE102485): (**A**) Focal adhesion, (**B**) EMC receptor interaction, (**C**) inflammation, and (**D**) apoptosis.

(A)

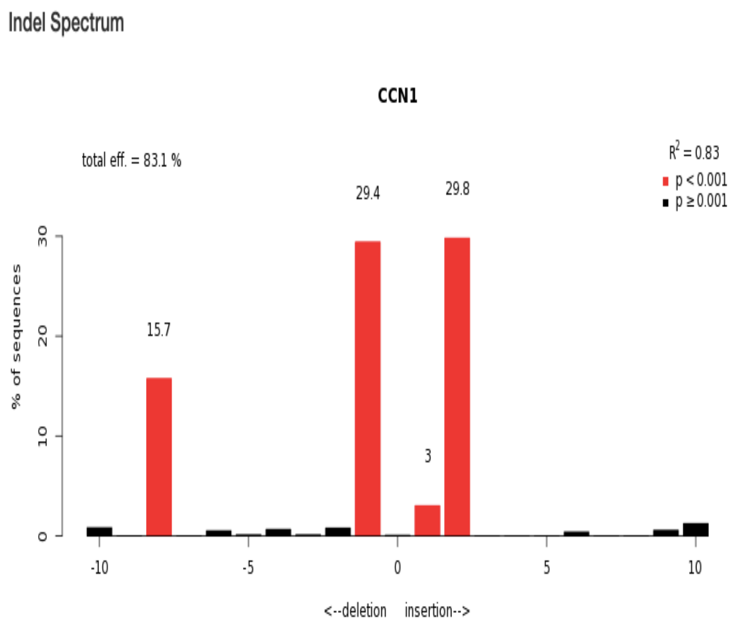

(B)

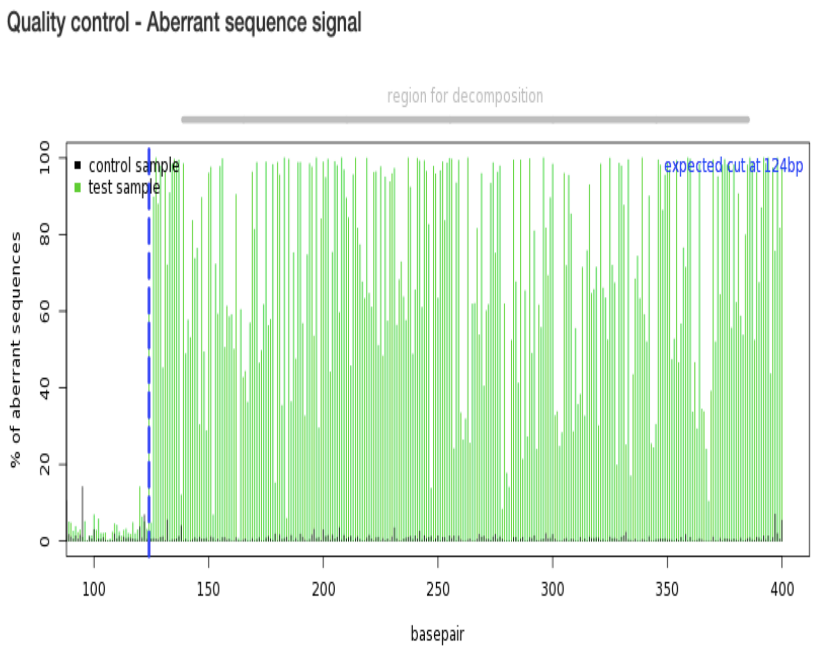

Figure S4. The construct of HRVECs$^{CCN1-/-}$ cell line. (**A**) Indel spectrum plotA depiction of the combination of trace models (indels) that can best explain the composite sequence trace in the experimental sample as determined by non-negative linear modelling; (**B**) sequence result show the efficient of CCN1 knock out.

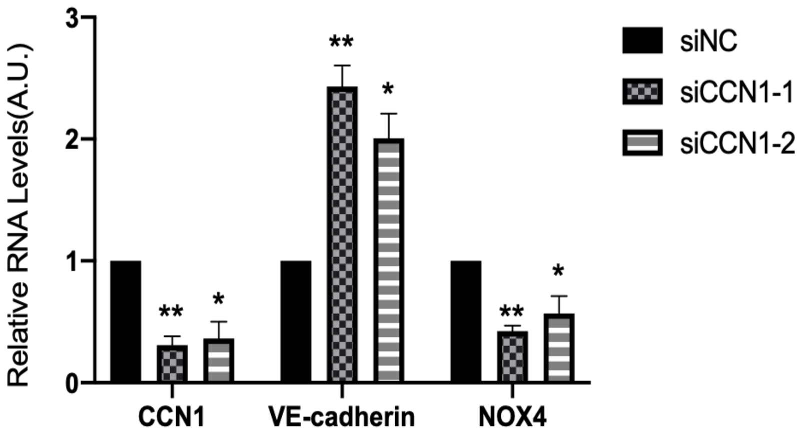

Figure S5. The Knock Down Efficiency of siRNA on the Expression of CCN1 and the regulate effect on the expression of VE-cadherin and NOX4.

Table S1. The main exclusion criterias for each group

Group	Age (year)	ACR(μg/min)	FFA
HC	45-55	<20	negtive
DMC	45-55	<20	negtive
DR	45-55	<20	positive

Table S2. the demographic characteristics of our enrolled cases

Charactors	HC(n=4)	T2DM(n=3)	P-value	DR(n=3)	P-value
**Age(years)**	50.25±2.36	47.33±12.22	NS	47.33±14.04	NS
**Gender(Female/Male)**	2/2	1/2	NS	1/2	NS
**BMI(kg/m²)**	NA	23.79±3.93	NA	25.87±1.08	NA
**SBP(mmHg)**	NA	126.33±5.51	NA	144.33±29.57	NA
**DBP(mmHg)**	NA	80.33±13.05	NA	84.00±2.65	NA
**HbA1c(%)**	5.45±0.29	9.57±2.14	0.01	11.00±2.05	0.00
**FPG(mmol/l)**	4.94±0.24	8.28±1.98	0.02	10.81±2.94	0.01
**FCP(nmol/l)**	NA	0.50±0.34	NA	0.50±0.27	NA
**0.5hCP(nmol/l)**	NA	0.58±0.19	NA	0.71±0.19	NA
**2hCP(nmol/l)**	NA	0.89±0.01	NA	1.29±0.39	NA
**K(mmol/l)**	NA	4.31±0.34	NA	4.32±0.37	NA
**Na(mmol/l)**	NA	140.67±1.53	NA	140.67±2.52	NA
**TC(mmol/l)**	5.99±0.95	3.81±0.72	0.21	5.61±0.94	0.63
**TG(mmol/l)**	1.13±0.27	1.28±0.83	0.75	2.35±0.52	0.01
**HDL-c(mmol/l)**	NA	0.97±0.06	NA	1.01±0.24	NA
**LDL-c(mmol/l)**	4.07±1.13	2.37±0.44	0.06	3.72±0.68	0.66
**BUN(mmol/l)**	NA	4.26±0.64	NA	5.58±1.72	NA
**CR(umol/l)**	NA	54.00±12.17	NA	42.67±5.51	NA
**ACR(mg/g)**	NA	8.22±3.91	NA	11.61±2.67	NA
**AST(U/L)**	NA	19.33±3.51	NA	27.67±10.26	NA
**ALT(U/L)**	NA	25.67±11.02	NA	26.33±10.41	NA
**TB(umol/l)**	NA	7.87±4.82	NA	10.70±2.26	NA
**DB(umol/l)**	NA	2.53±1.71	NA	2.10±0.26	NA
**IB(umol/l)**	NA	5.33±3.17	NA	8.60±2.20	NA

NOTE: NA is short for not access, NS is short for no significance.
